# Muscle- and Mode-Specific Responses of the Forearm Flexors to Fatiguing, Concentric Muscle Actions

**DOI:** 10.3390/sports4040047

**Published:** 2016-09-30

**Authors:** Ethan Hill, Terry Housh, Cory Smith, Richard Schmidt, Glen Johnson

**Affiliations:** Department of Nutrition and Health Sciences, University of Nebraska-Lincoln, Lincoln, NE 68505, USA; thoush1@unl.edu (T.H.); csmith@unl.edu (C.S.); rschmidt1@unl.edu (R.S.); gojohnson22@gmail.com (G.J.)

**Keywords:** muscle fatigue, isokinetic, EMG, MMG

## Abstract

Background: Electromyographic (EMG) and mechanomyographic (MMG) studies of fatigue have generally utilized maximal isometric or dynamic muscle actions, but sport- and work-related activities involve predominately submaximal movements. Therefore, the purpose of the present investigation was to examine the torque, EMG, and MMG responses as a result of submaximal, concentric, isokinetic, forearm flexion muscle actions. Methods: Twelve men performed concentric peak torque (PT) and isometric PT trials before (pretest) and after (posttest) performing 50 submaximal (65% of concentric PT), concentric, isokinetic (60°·s^−1^), forearm flexion muscle actions. Surface EMG and MMG signals were simultaneously recorded from the biceps brachii and brachioradialis muscles. Results: The results of the present study indicated similar decreases during both the concentric PT and isometric PT measurements for torque, EMG mean power frequency (MPF), and MMG MPF following the fatiguing workbout, but no changes in EMG amplitude (AMP) or MMG AMP. Conclusions: These findings suggest that decreases in torque as a result of fatiguing, dynamic muscle actions may have been due to the effects of metabolic byproducts on excitation–contraction coupling as indicated by the decreases in EMG MPF and MMG MPF, but lack of changes in EMG AMP and MMG AMP from both the biceps brachii and brachioradialis muscles.

## 1. Introduction

Surface electromyography (EMG) records and quantifies the action potentials that activate skeletal muscle fibers [1]. The amplitude (AMP) of the EMG signal is generated by the summation of the action potential trains from the active motor units and is influenced by the number of active motor units, their firing rates, and synchronization [1,2]. The power spectrum of the EMG signal is, in part, determined by average muscle fiber action potential conduction velocity [3] and the shape of the action potential waveforms [4]. Mechanomyography (MMG) is a non-invasive technique that provides information related to muscle function, which is unique compared with EMG. Gordon and Holbourn (1948) [5] described the MMG signal as the mechanical counterpart of motor unit activity as measured by EMG. MMG quantifies the lateral oscillations of activated muscle fibers that are generated by (a) gross lateral movement of the muscle at the initiation of a contraction generated by the non-simultaneous activation of muscle fibers; (b) smaller subsequent lateral oscillations generated at the resonance frequency of the muscle; and (c) dimensional changes of the active fibers [6]. It has been suggested that the amplitude of the MMG signal is related to motor unit recruitment and that the frequency content of the MMG signal is qualitatively related to the motor unit firing rate [6,7,8]. A number of anatomical, physiological, and non-physiological factors can affect the time and frequency domain parameters of the EMG signal including subcutaneous tissue layer, environmental noise, wave cancellation, and electrode placement [1,9,10,11]. In addition, the MMG signal can be influenced by muscle temperature, muscle stiffness, mass, the viscosity of the intracellular and extracellular fluids, and intramuscular fluid pressure [6,12,13].

A unique application of EMG and MMG involves the assessment of muscle fatigue. Muscle fatigue has been described as “an acute impairment of performance that includes both an increase in perceived effort necessary to exert a desired force and an eventual inability to produce this force” [14] (p. 1631) and “can occur despite continued and successful performance of a submaximal task” [15] (p. 133). Previous investigations have examined muscle fatigue as assessed by changes in torque, EMG, and MMG during maximal and submaximal, isometric muscle actions [6,16,17,18,19,20,21]. For example, following maximal, intermittent, isometric, leg extension muscle actions, Camic et al. (2013) [16] reported decreases in isometric peak torque (PT), EMG AMP, EMG mean power frequency (MPF), and MMG MPF, but no change in MMG AMP from the vastus lateralis. During fatiguing, submaximal, intermittent isometric muscle actions, Orizio (1993) [6] reported intensity-specific responses for MMG AMP during sustained, isometric, forearm flexion muscle actions at submaximal intensities of 20%–80% of isometric PT. Furthermore, Seghers and Spaepen (2004) [20] reported a decrease and no change in EMG median frequency from the biceps brachii (BB) during intermittent, isometric, forearm flexion muscle actions at submaximal intensities of 25% and 50% of isometric PT, respectively.

A number of investigations [18,22,23,24,25,26,27] have also examined the responses as a result of maximal, dynamic movements, but a limited number of investigations [28] have examined the effects of fatiguing, submaximal, dynamic workbouts on EMG or MMG responses. For example, Camic et al. (2013) [16] examined the effects of 30 repeated, maximal, concentric muscle actions at 30°·s^−1^ and reported decreases in concentric PT, EMG AMP, EMG MPF, MMG AMP, and MMG MPF from the vastus lateralis. During fatiguing, maximal, eccentric workbouts at velocities of 60°·s^−1^, 120°·s^−1^, and 180°·s^−1^, Hill et al. (2015) [27] reported no changes in eccentric torque, but isometric PT decreased 17.9%, 16.1%, and 8.9%, respectively. In addition, Perry-Rana et al. (2003) [24] reported muscle-specific EMG and MMG responses for the vastus lateralis, vastus medialis, and rectus femoris as a result of fatiguing, maximal, eccentric muscle actions at 120°·s^−1^. Together, these investigations have shown that fatigue-related changes in the time and frequency domain parameters of EMG and MMG signals can be affected by the mode, intensity, and velocity of muscle action employed (isometric vs. concentric vs. eccentric), as well as the muscles groups involved.

Most sport- and work-related activities involve predominately submaximal, dynamic movements. Thus, understanding the fatigue-related neuromuscular responses associated with submaximal, dynamic muscle actions may have implications regarding work- and sport-related interventions to improve athletic performance, reduce sport- and work-related musculoskeletal injuries, or both [29,30,31,32,33]. Therefore, the purpose of the present investigation was to examine the torque, EMG, and MMG responses as a result of submaximal, concentric, isokinetic, forearm flexion muscle actions. Based on previous investigations [20,27], we hypothesized that concentric PT, isometric PT, EMG MPF, MMG AMP, and MMG MPF would decrease as a result of the 50 submaximal, concentric muscle actions, while EMG AMP would remain unchanged.

## 2. Results

### 2.1. Concentric PT and Isometric PT Responses

**Figure 1:**
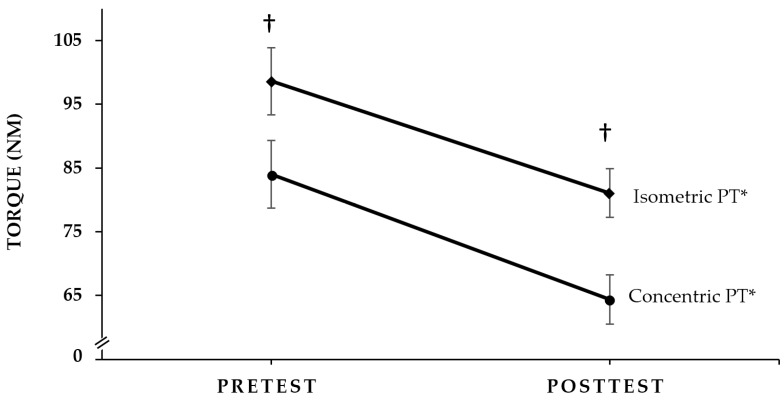
Pretest and posttest values (±SEM) for concentric peak torque (PT) and isometric PT. * Significant at *p* ≤ 0.05 for pretest > posttest. **†** Significant at *p* ≤ 0.05 for concentric PT < isometric PT.

Figure 1 shows the pretest and posttest concentric PT and isometric PT responses. There was no significant Mode × Time interaction for torque (*p* = 0.347, $\eta_{p}^{2}$ = 0.081). There were, however, significant main effects for Mode (isometric PT > concentric PT; *p* < 0.001, $\eta_{p}^{2}$ = 0.872) and Time (pretest > posttest; *p* < 0.001, $\eta_{p}^{2}$ = 0.841). Thus, torque decreased during both the concentric PT (23.3%) and isometric PT (17.8%) muscle actions as a result of the fatiguing workbout, and concentric PT was less than isometric PT at pretest and posttest.

### 2.2. Concentric PT and Isometric PT Neuromuscular Responses

**Figure 2:**
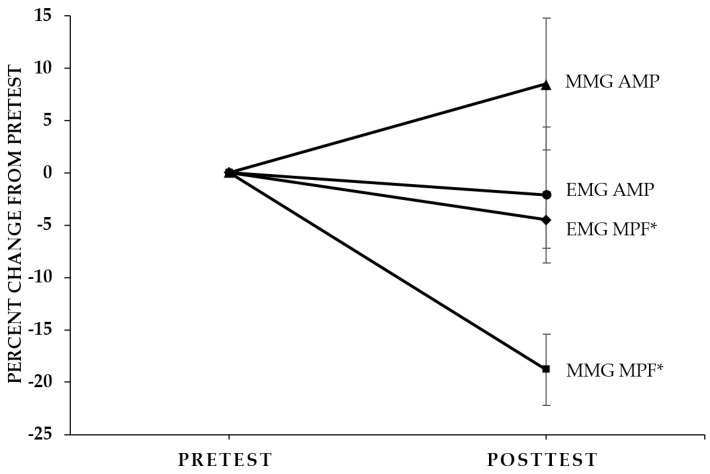
Pretest and posttest values (±SEM) expressed as percentage changes from pretest (collapsed across Muscle (biceps brachii and brachioradialis) and Mode (concentric peak torque and isometric peak torque)) for electromyographic amplitude (EMG AMP), EMG mean power frequency (EMG MPF), mechanomyographic amplitude (MMG AMP), and MMG MPF. * Significant at *p* ≤ 0.05 for pretest > posttest.

EMG AMP: There were no significant 3- (Muscle × Mode × Time, *p* = 0.325, $\eta_{p}^{2}$ = 0.088) or 2-way interactions (Time × Mode, *p* = 0.382, $\eta_{p}^{2}$ = 0.070; Muscle × Mode, *p* = 0.165, $\eta_{p}^{2}$ = 0.167; Muscle × Time, *p* = 0.065, $\eta_{p}^{2}$ = 0.276) for normalized EMG AMP. In addition, there were no significant main effects for Mode (*p* = 0.435, $\eta_{p}^{2}$ = 0.056), Time (*p* = 0.701, $\eta_{p}^{2}$ = 0.014), or Muscle (*p* = 0.179, $\eta_{p}^{2}$ = 0.158). Thus, there was no change in EMG AMP as a result of the fatiguing workbout (Figure 2).

EMG MPF: There were no significant 3- (Muscle × Mode × Time, *p* = 0.185, $\eta_{p}^{2}$ = 0.154) or 2-way interactions (Time × Mode, *p* = 0.115, $\eta_{p}^{2}$ = 0.210; Muscle × Mode, *p* = 0.567, $\eta_{p}^{2}$ = 0.031; Muscle × Time, *p* = 0.891, $\eta_{p}^{2}$ = 0.002) for normalized EMG MPF. There was, however, a significant main effect for Time (pretest > posttest; *p* = 0.045, $\eta_{p}^{2}$ = 0.317), collapsed across Muscle and Mode, but no significant main effects for Mode (*p* = 0.745, $\eta_{p}^{2}$ = 0.010) or Muscle (*p* = 0.810, $\eta_{p}^{2}$ = 0.005). Thus, EMG MPF decreased as a result of the fatiguing workbout (Figure 2).

MMG AMP: There was a significant 3-way (*p* = 0.042, $\eta_{p}^{2}$ = 0.324) interaction for normalized MMG AMP that was decomposed into separate 2-way ANOVAs (Mode × Time) for each muscle. There were no significant Mode × Time interactions for the BB (*p* = 0.172, $\eta_{p}^{2}$ = 0.686) or the BR (*p* = 0.053, $\eta_{p}^{2}$ = 0.299). There were, however, significant main effects for Mode from the BB (concentric PT > isometric PT; *p* = 0.004, $\eta_{p}^{2}$ = 0.578) and BR (concentric PT > isometric PT; *p* = 0.001, $\eta_{p}^{2}$ = 0.755), but no main effects for Time from the BB (*p* = 0.112, $\eta_{p}^{2}$ = 0.538) or BR (*p* = 0.827, $\eta_{p}^{2}$ = 0.005). Thus, MMG AMP was greater during the concentric PT than the isometric PT measurements.

MMG MPF: There were no significant 3- (Muscle × Mode × Time, *p* = 0.466, $\eta_{p}^{2}$ = 0.049) or 2-way interactions (Time × Mode, *p* = 0.925, $\eta_{p}^{2}$ = 0.001; Muscle × Mode, *p* = 0.402, $\eta_{p}^{2}$ = 0.065; Muscle × Time, *p* = 0.951, $\eta_{p}^{2}$ < 0.001) for normalized MMG MPF. There was, however, a significant main effect for Time (pretest > posttest; *p* < 0.001, $\eta_{p}^{2}$ = 0.715), collapsed across Muscle and Mode, but no significant main effects for Mode (*p* = 0.674, $\eta_{p}^{2}$ = 0.017) or Muscle (*p* = 0.877, $\eta_{p}^{2}$ = 0.002). Thus, MMG MPF decreased as a result of the fatiguing workbout (Figure 2).

## 3. Discussion

### 3.1. The Effects of the Submaximal, Dynamic Workbout

The purpose of the present investigation was to examine the torque, EMG, and MMG responses as a result of submaximal, concentric, isokinetic, forearm flexion muscle actions. The findings of the present study indicated that there were no mode-specific effects for torque, EMG AMP, EMG MPF, or MMG MPF. There was, however, a mode-specific effect for MMG AMP which was greater during the concentric PT than the isometric PT measurements. Furthermore, consistent with our hypothesis and previous investigations [20,27], concentric PT, isometric PT, EMG MPF, and MMG MPF decreased, while EMG AMP remained unchanged. Unlike our hypothesis, however, there was no change in MMG AMP.

### 3.2. Torque

The results of the present study indicated that there was no mode-specific effect for the decline in maximal torque following the submaximal, fatiguing workbout. That is, even though the fatiguing workbout involved only concentric, isokinetic muscle actions, there was no difference in the percent declines in concentric PT (23.3%) and isometric PT (17.8%). These findings were consistent with those of Camic (2011) [34] who reported a 20.3% decline in concentric PT and a 16.5% decline in isometric PT following 30 maximal, concentric, isokinetic, leg extension muscle actions in women. Thus, the results of the present study, in conjunction with those of Camic (2011) [34], indicated that, for both genders, there were no mode-specific declines in maximal torque and similar decreases in concentric PT and isometric PT following submaximal or maximal, fatiguing, concentric workbouts.

### 3.3. Pretest Versus Posttest EMG and MMG Responses

There were no muscle- or mode-specific patterns of differences in pretest versus posttest neuromuscular responses as a result of the submaximal, fatiguing workbout in the present study, but collapsed across Time and Muscle MMG AMP was greater during the concentric PT than the isometric PT measurements. The greater MMG AMP values during the concentric PT than isometric PT measurements may have been due to the dynamic nature of the muscle action [8,35]. It is also possible that the lower concentric PT values (84.0 and 64.4 Nm at pretest and posttest, respectively), compared with the isometric PT (98.6 and 81.1 Nm at pretest and posttest, respectively) values, were associated with less muscle stiffness, greater muscle compliance, or both, which resulted in less restriction of the lateral oscillations of the activated muscle fibers and, therefore, greater MMG AMP values [8,35].

Fatigue is typically associated with decreases in EMG MPF which reflect the effects of metabolic byproducts on muscle fiber action potential conduction velocity [1,2]. Thus, the pretest versus posttest decrease in EMG MPF in the present study, along with the decreases in concentric PT and isometric PT, supported the fatiguing nature of the workbout. Furthermore, the pretest versus posttest decreases in concentric PT and isometric PT, without changes in EMG AMP and MMG AMP suggested excitation–contraction coupling failure. That is, the buildup of metabolic byproducts such as lactate, inorganic phosphate, and ammonia interfere with contractile properties of the activated muscle fibers [36,37,38,39,40]. Although there is some disagreement [41,42], the effect of lactate and inorganic phosphate accumulation on force production may be due to the effects of calcium release and reuptake by the sarcoplasmic reticulum, actin-myosin binding affinity, troponin-calcium binding affinity, ATP breakdown via ATPase, and ATP production in the metabolic pathways [39]. In addition, ammonia accumulation during exercise can adversely affect action potential propagation [37,40]. Thus, it is possible that the fatigue-induced buildup of metabolic byproducts caused excitation–contraction coupling failure and contributed to the decreases in concentric PT and isometric PT.

In the present study, the decrease in global motor unit firing rate (MMG MPF) may have been related to the effects of muscle wisdom [43,44]. Muscle wisdom is a motor unit activation strategy characterized by decreased muscle relaxation times and motor neuron discharge rates as well as greater fusion of motor unit twitches to optimize force production [43,44,45,46,47]. The applicability of muscle wisdom during dynamic muscle actions, however, has been questioned [44]. In addition, the decrease in global motor unit firing rate (MMG MPF) without changes in EMG AMP or MMG AMP did not support the suggestion of Orizio (1993) [6] regarding fatigue-related de-recruitment of motor units [6], but may have been due to synchronization. Specifically, EMG AMP is a function of motor unit recruitment, firing rate, and synchronization [1,2,48]. Although there is some disagreement regarding the occurrence of synchronization [49,50], the lack of change in EMG AMP despite decreases in motor unit firing rate (MMG MPF) and no change in motor unit recruitment (MMG AMP) suggested that motor unit synchronization, which results in a more efficient and synchronous activation of motor units to optimize force production [1,51,52,53,54,55], compensated for the decrease in firing rate.

## 4. Materials and Methods

### 4.1. Subjects

Thirteen men volunteered to participate in this investigation, but 1 subject was unable to complete all testing procedures (*n* = 12; mean age ± SD = 22.6 ± 2.2 years; body weight = 84.0 ± 8.3 kg; height = 178.6 ± 8.3 cm). The subjects regularly participated in resistance training (8.1 ± 2.2 h per week) and had no known cardiovascular, pulmonary, metabolic, muscular, or coronary heart disease, or regularly used prescription medication. The subjects visited the laboratory on 2 occasions separated by at least 48-h and were instructed not to perform upper body exercise 48-h prior to each visit. The study was approved by the University Institutional Review Board for Human Subjects, and all subjects completed a health history questionnaire and signed written informed consent prior to testing.

### 4.2. Procedures

Familiarization (Visit 1): The first laboratory visit consisted of an orientation session to familiarize the subjects with the testing protocols. During the orientation, the subjects performed submaximal and maximal concentric, isokinetic (60°·s^−1^) and isometric muscle actions of the forearm flexors. The subjects visually tracked torque production using real-time torque displayed on a computer monitor programmed using LabVIEW 13.0 software (National Instruments, Austin, TX, USA) and practiced performing concentric, isokinetic muscle actions at 65% of concentric PT.

Determination of Concentric PT and Isometric PT: During Visit 2, the subjects performed a warm-up consisting of 10–15 submaximal (approximately 50%–75% of PT), concentric, isokinetic muscle actions of the dominant (based on throwing preference) forearm flexors at 60°·s^−1^ on a calibrated Cybex II dynamometer. After 2 min of rest, the subjects randomly performed 5 concentric PT and 5 isometric PT trials [56]. The concentric muscle actions were performed at 60°·s^−1^ through a 90° range of motion (from 170° to 80°), and the isometric muscle actions were performed for 4 s at an elbow joint angle of 115° where 180° corresponded to full extension [57,58,59,60]. The highest values were selected as the pretest concentric PT and isometric PT, respectively.

Submaximal, Concentric, Isokinetic Muscle Actions: Following the determination of the pretest concentric PT and pretest isometric PT, the subjects performed 50 submaximal (65% of their pretest concentric PT), concentric, isokinetic muscle actions at 60°·s^−1^ followed by passive forearm extension at 60°·s^−1^ that were assisted by the investigator. Real-time torque was displayed on a computer monitor. In addition, a light bulb indicated the start and end of each repetition, which was displayed on the same computer monitor as the real-time torque. For analyses, only subjects that maintained 65% (±5%) of PT during all 50 submaximal, concentric muscle actions were used. Thus, only the data from 12 of the 13 subjects were analyzed. After completing the 50 submaximal, concentric muscle actions, the subjects randomly performed 5 posttest concentric PT and 5 posttest isometric PT trials using the same procedures as the pretest.

Electrode and Accelerometer Placements: During Visit 2, bipolar (30 mm center-to-center) surface EMG electrode (circular 4-mm diameter silver–silver chloride, BIOPAC Systems, Inc., Santa Barbara, CA, USA) arrangements were placed on the dominate arm over the BB and BR muscles according to the recommendations of Barbero et al. (2012) [61]. The reference electrode was placed over the acromion process. Prior to each electrode placement, the skin was shaved, carefully abraded, and cleaned with alcohol. The MMG signals from the BB and BR were detected using accelerometers (Entran EGAS FT 10, dimensions: 1.0 × 1.0 × 0.5 cm^3^, mass: 1.0 g, sensitivity: 651.6 and 624.3 mV/g) that were placed between the proximal and distal EMG electrodes of each of the bipolar arrangement using double-sided adhesive tape.

Signal Processing: The raw EMG and MMG signals were digitized at 1000 Hz with a 32-bit analog-to-digital converter (Model MP100, Biopac Systems, Inc., Santa Barbara, CA, USA) and stored in a personal computer (ATIV Book 9 Intel Core i7 Samsung Inc., Dallas, TX, USA) for subsequent analyses. The EMG signals were amplified (gain: ×1000) using differential amplifiers (EMG 100, Biopac Systems, Inc., Santa Barbara, CA, USA). The EMG and MMG signals were digitally bandpass filtered (fourth-order Butterworth, zero-phase shift) at 10–500 Hz and 5–100 Hz, respectively. All signal processing was performed using custom programs written with the LabVIEW programming software. The EMG (µV root-mean-square, µVrms) and MMG (m·s^−2^) AMP and MPF (Hz) values for the concentric and isometric muscle actions were calculated for the middle third of each contraction. Thus, during the concentric and isometric muscle actions, signal epochs of 0.50 s and 1.33 s were used, respectively, to calculate the AMP and MPF values of the EMG and MMG signals. These portions of the signals were selected to avoid the acceleration and deceleration phases that are typical of isokinetic dynamometers [62] and to avoid the initial gross lateral movement of the muscle at the onset of muscle contraction [6]. For the MPF analyses, each data segment was processed with a Hamming window and the Discrete Fourier transform (DFT) algorithm [63,64]. The MPF was selected to represent the power spectrum in accordance with the recommendations of Hermens et al. (2000) [65]. All EMG and MMG analyses were performed using normalized (to pretest isometric PT) values (Table 1).

### 4.3. Statistical Analyses

A 2 (Mode (concentric PT, isometric PT)) × 2 (Time (pretest, posttest)) repeated measures ANOVA was used to analyze the absolute concentric PT and isometric PT. In addition, separate 2 (Muscle (BB, BR)) × 2 (Mode (concentric PT, isometric PT)) × 2 (Time (pretest, posttest)) repeated measures ANOVAs were used to analyze the normalized (to values during the pretest isometric PT) EMG AMP, EMG MPF, MMG AMP, and MMG MPF values. Partial eta squared effect sizes ($\eta_{p}^{2}$) were calculated for each ANOVA. Significant 3-way interactions were decomposed with follow-up repeated measures ANOVAs, and significant 2-way interactions were decomposed with follow-up, Bonferroni-corrected dependent samples t-tests. In addition, Greenhouse–Geisser corrections were applied when the assumption of sphericity was not met according to Maulchy’s test of sphericity. All statistical analyses were performed using IBM SPSS v. 21 (Armonk, NY, USA), and an alpha of *p* ≤ 0.05 was considered statistically significant.

## 5. Conclusions

In summary, the results of the present study indicated that there were no mode-specific declines in maximal torque and similar decreases in concentric PT and isometric PT following the submaximal fatiguing, concentric workbout. In addition, there were no muscle- or mode-specific patterns of differences in neuromuscular responses as a result of the fatiguing workbout. MMG AMP, however, was greater during the concentric PT than the isometric PT measurements, which may have been due to the dynamic nature of the muscle action, muscle stiffness, muscle compliance, or a combination thereof. As a result of the fatiguing workbout, there were decreases from pretest to posttest for EMG MPF and MMG MPF, but no changes in EMG AMP or MMG AMP (Figure 2). The pretest versus posttest decreases in concentric PT and isometric PT, without changes in EMG AMP and MMG AMP suggested excitation–contraction coupling failure. The decrease in global motor unit firing rate (MMG MPF) without changes in EMG AMP or MMG AMP may have been related to the effects of muscle wisdom, motor unit synchronization, or both.

## Figures and Tables

**Table 1:** Descriptive data (means ± SD) for the normalized pretest and posttest neuromuscular responses from the biceps brachii (BB) and brachioradialis (BR) during the concentric peak torque (PT) and isometric PT muscle actions.

Neuromuscular Parameters		Concentric PT		Isometric PT	
		BB	BR	BB	BR
**EMG AMP (μV)**	Pretest	1.08 ± 0.20	1.04 ± 0.16	1.00 ± 0.00	1.00 ± 0.00
	Posttest	1.12 ± 0.40	0.90 ± 0.16	1.05 ± 0.37	0.97 ± 0.17
**EMG MPF (Hz)**	Pretest	1.05 ± 0.27	1.01 ± 0.12	1.00 ± 0.00	1.00 ± 0.00
	Posttest	0.93 ± 0.24	0.97 ± 0.13	1.03 ± 0.27	0.96 ± 0.15
**MMG AMP (m·s^−2^)**	Pretest	1.23 ± 0.42	1.21 ± 0.32	1.00 ± 0.00	1.00 ± 0.00
	Posttest	1.42 ± 0.46	1.43 ± 0.38	1.12 ± 0.27	0.81 ± 0.22
**MMG MPF (Hz)**	Pretest	1.23 ± 0.49	1.13 ± 0.38	1.00 ± 0.00	1.00 ± 0.00
	Posttest	1.12 ± 0.35	1.19 ± 0.27	0.98 ± 0.33	0.95 ± 0.31
